# A comparative study of worm-sludge treatment reed bed planted with *Phragmites australis* and *Arundo donax* in the Mediterranean region

**DOI:** 10.1007/s11356-024-34632-9

**Published:** 2024-08-08

**Authors:** Amir Gholipour, Rita Fragoso, Ana Galvão, Elizabeth Duarte

**Affiliations:** 1 LEAF–Linking Landscape, Environment, Agriculture and Food, School of Agriculture (ISA), University of Lisbon, Tapada da Ajuda, 1349-017 Lisbon, Portugal; 2 LEAF–Linking Landscape, Environment, Agriculture and Food, Associate Laboratory TERRA, School of Agriculture (ISA), University of Lisbon, Tapada da Ajuda, 1349-017 Lisbon, Portugal; 3 CERIS, Technical University of Lisbon (IST), Av. Rovisco Pais, 1049-001 Lisbon, Portugal

**Keywords:** Sludge management, Constructed wetland, Nature-based solutions, Sludge dewatering, *Eisenia fetida*

## Abstract

Sludge treatment reed bed planted (STRB) with *Phragmites australis* (*P.australis*) and *Arundo donax* (*A.donax*) was assessed in the presence of *Eisenia fetida* under control condition during the dry season. Worm-planted units were fed with mixed sewage sludge (dry and volatile solids of 29.44 g DS.L^−1^ and 24.23 g VS.L^−1^). Sludge loading rates (SLR) of 50, 60, and 70 kg DS m^−2^ year^−1^ were examined to assess dewatering efficiency. Surface layers in units with *P.australis* and *A.donax* achieved DS of 80 and 81% at a loading rate of 50 kg DS m^−2^ year^−1^, while their subsurface DS were 41 and 25%, respectively. Units with *A.donax* experienced plant loss when subjected to SLR exceeding 60 kg DS m^−2^ year^−1^. More than 10 cm of residual sludge accumulated on the top of units after a 2-month final rest. Evapotranspiration was greater in the unit with *P.australis* (5.23 mm day^−1^) compared to the unit with *A.donax* (4.24 mm day^−1^) while both were fed with 70 kg DS m^−2^ year^−1^. Water loss contributions from residual sludge layer, drained water, and evapotranspiration were 3, 46, and 51%, respectively. Units with *P.australis* indicated 20% higher water loss compared to units with *A.donax*. Although the drained water quality improved over time, it did not meet standard limits. The residual sludge layer contained macro and micronutrients, and heavy metals with a significant elemental order of N > Ca > P > S > mg > K (N:P:K = 31:8:1), Fe > Na > B > Mn > Mo and Zn > Cr > Cu > Pb > Ni > Cd. Overall, STRB could be a sustainable alternative technology to conventional sewage sludge management techniques.

**Graphical Abstract:**

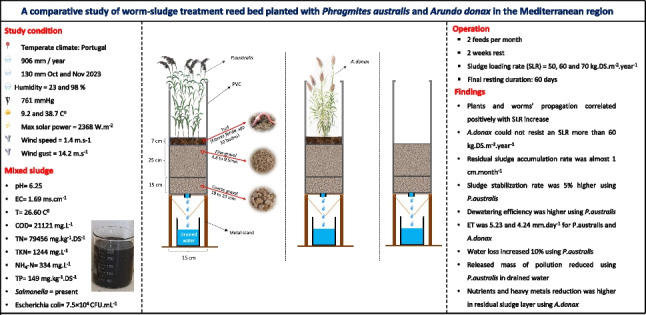

**Supplementary Information:**

The online version contains supplementary material available at 10.1007/s11356-024-34632-9.

## Introduction

The significance of sanitation in modern cities, extremely underscored during the pandemic, stands as a critical priority (Hannah et al. 2020). The sixth sustainable development goal, focusing on clean water and sanitation, is pivotal not just as a fundamental right but also as a critical action, especially in the face of resource scarcities such as water and nutrients (Tortajada and Biswas 2018). Urban development has magnified the urgency of sanitation, especially concerning wastewater treatment (van der Merwe and Simha 2023). The current scarcity of resources, particularly food and water, demands an exploration of alternative resources. Nutrient depletion stands as a prominent global challenge exacerbating concerns for productive agriculture. Dwindling phosphorus availability in agricultural lands poses a threat to soil fertility, directly impacting crop yield and quality (Marschner and Rengel 2023). The prevailing linear economy, starting from raw material extraction and culminating in disposal, is no longer a sustainable approach; therefore, embracing a circular economy offers a viable alternative paradigm (Morseletto 2023). In a circular economy, resources are recovered and reused, which is illustrated in sanitation by the treatment of wastewater aiming the recovery of valuable resources in sewage sludge (Mannina et al. 2023). Sewage sludge holds high amount of organic and inorganic contents (Gholipour et al. 2022); however, there are still challenges in its recovery, primarily due to high costs and environmental impacts of conventional techniques (Daee et al. 2019; Tarpani and Azapagic 2018). Conventional processes contribute to climate change, prompting the need for a paradigm shift (Chang et al. 2023). Global warning potential of technological processes were found positive in previous studies compared to nature-based solutions (NBS) (Uggetti et al. 2011; Zhuang et al. 2022). NBS, like sludge treatment reed red (STRB), offers an eco-friendly alternative to energy-intensive solutions such as centrifugation (Nielsen 2023). STRB, as an application of treatment wetland, employs sand and gravel media planted with common reeds like *Phragmites australis* (*P.australis*) (Gholipour et al. 2020; Gholipour and Stefanakis 2021). STRB significantly reduces energy consumption compared to energy-intensive solutions, requiring minimal energy input. NBS proves high performance in reducing operation, maintenance, and capital costs, outshining energy-intensive solutions (Chang et al. 2023; Megyesi et al. 2024). Energy-intensive solutions also fail to provide a reusable by-product and often need further treatment for reuse purposes, contributing to environmental degradation and a positive global warning potential (Zhuang et al. 2022). While STRB stands as a promising NBS, offering numerous advantages over conventional techniques, it requires improvement, particularly in reducing land demand and enhancing the quality of residual sludge (Gholipour et al. 2023).

Previous studies have explored advancements in typical STRB, introducing variations like electro-STRB (E-STRB or STEW), worm-STRB (W-STRB), and intensified-STRB (I-STRB) (Chen et al. 2016; Chen and Hu 2019; Hu et al. 2020; Hu and Chen 2018; Plestenjak et al. 2021; Wang et al. 2021; Zhong et al. 2021). E-STRB aimed not only at sludge dewatering but also at generating power from organic matter via microbial fuel cells which yielded 60 mW m^−3^ in previous studies (Saeed et al. 2022). W-STRB primarily addresses land demand through an enhancement in sludge loading rate (SLR) up to 120 kg dry solid m^−2^ year^−1^ in tropical climate (Chen et al. 2016; Hu et al. 2020). Worms were introduced also to enhance residual sludge stabilization and mineralization (Calderón-Vallejo et al. 2015). Meanwhile, I-STRB involves an aeration system injecting air into the media to augment dewatering efficiency and organic matter decomposition (Plestenjak et al. 2021). In contrast, typical STRB relies on passive oxygen intake through plant roots or ventilation systems (Kołecka et al. 2018; Nielsen 2007; Stefanakis and Tsihrintzis 2011; Uggetti et al. 2009). Moreover, the development of STRB variants has involved testing different plant species in the dewatering process of which *P.australis* was the most common species used while *Typha latifolia*, *Typha angustifolia*, *Canna indica*, *Cynodon dactylon pers*, *Echinochloa pyramidalis*, and *Cyperus papyrus* were the other species tested (Gholipour et al. 2022). Native plant species were anticipated to exhibit high efficiency, especially concerning dewatering efficiency, reduction in contaminants in STRB-drained water, and higher stabilization (Nielsen 2011). STRB played a crucial role in effectively removing various pollutants from residual sludge and drained water including nitrogen components, phosphorus, heavy metals, pharmaceuticals, personal care products, organic compounds, micropollutants, and greenhouse gases (Chen and Hu 2019; Liang et al. 2021; Ma et al. 2020; Nielsen 2023; Stefanakis and Tsihrintzis 2012; Zhang et al. 2016).

The typical STRB has been tested across different climates, whereas E-STRB, W-STRB, and I-STRB have mostly undergone controlled testing in tropical (E-STRB and W-STRB) and temperate (I-STRB) climates (Hu and Chen 2018; Plestenjak et al. 2021; Wang et al. 2021). The process of sewage sludge dewatering with STRB relies on evapotranspiration (ET), and drainage which are highly influenced by climate (Brix 2017). Consequently, there is a pressing need to explore STRB variants in various climates. SLR commonly serves as a design parameter, and previous studies have showcased varying SLRs for E-STRB, W-STRB, and I-STRB, often enhanced in drier climates (Gholipour et al. 2022). Previous studies in China, Brazil, and Oman have employed *P.australis* in controlled conditions for W-STRB without assessing the impact of seasonal variations on dewatering efficiency (Al-Rashdi et al. 2024; Calderón-Vallejo et al. 2015; Hu and Chen 2018; Ma et al. 2023). On the contrary, our preliminary study in Portuguese temperate climate analyzed the effect of seasonal variations on W-STRB efficiency (Gholipour et al. 2024b, a). W-STRB was assessed for the dewatering of mixed sludge from an urban wastewater treatment plant (WWTP) where an SLR of 43.5 kg DS m^−2^ year^−1^ was applied. This paper serves as a follow-up of Gholipour et al. (2024b), focusing on assessing the cooperative effect of worms and plants under elevated SLR ranges of 50, 60, and 70 kg DS m^−2^ year^−1^. This study was conducted in the Horto greenhouse at the School of Agriculture of the University of Lisbon (ISA) and compares the W-STRB system planted with *P.australis* and *Arundo donax* (*A.donax*) concerning dewatering efficiency, water balance, drained water quality, and final residual sludge quality. The findings from this study can assist researchers and engineers who are considering the adoption of this NBS as a diversion technology applied to mixed sludge dehydration in similar climate conditions.

## Materials and methods

### Study area and experimental setup

The experiment was conducted in Horto greenhouse facility, ISA, University of Lisbon, Portugal, 2023 (38° 42′ 28.9″ N 9° 11′ 06.4″ W). The research area was without protective cover for the bench-scale mesocosm, resulting in the mesocosms being subjected to precipitation and ambient temperature. The experiment proceeded to evaluate the W-STRB system in Portugal, building upon our previous investigation conducted in Beirolas over the course of 1 year, which accounted for seasonal variations (Gholipour et al. 2024b). Our findings from the Beirolas experiment indicated that the W-STRB system reached maturity after 6 months of operation. In the current study conducted in Horto, we specifically focused on the initial 6-month period to avoid redundant repetitions, considering Horto as a follow-up to the Beirolas experiment. Meteorological data were recorded through an onsite weather station including maximum, minimum, and average air temperature (°C), solar power (W m^−2^), humidity (%), wind speed (m s^−1^), and precipitation (mm).

**Fig. 1:**
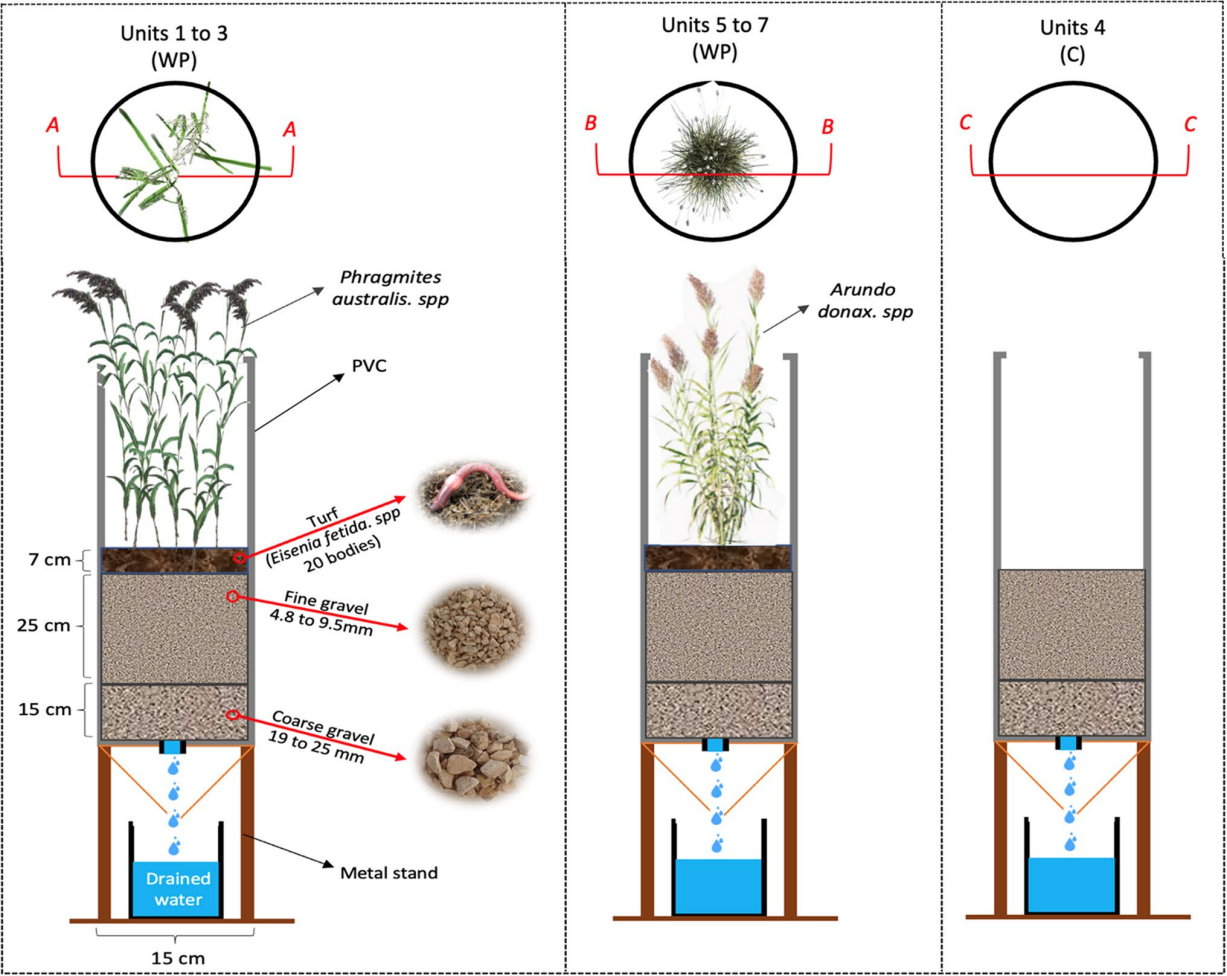
Pilot study configuration

The experiment included seven units built in 150 mm diameter (60 cm height) PVC pipe (Fig. 1). Units 1 to 3 were planted with *P.australis* (WP1, WP2, and WP3); units 5 to 7 with *A.donax* (WP5, WP6, and WP7); and all units included worms. Unit 4 served as a control, without plants and worms. All units were filled with two layers of gravel containing a drainage layer (15 cm of coarse gravels 19 to 25 mm, and 38% porosity) and a transition layer (25 cm of fine gravels, 4.8 to 9.5 mm, and 42% porosity). Except for the control unit, a turf layer (Siro 30) was added on the top to host 20 bodies of *Eisenia fetida*. One tuft of reeds was planted in WP units (Brix 2017), and worms were inserted a week before feeding to acclimatize into the turf layer (Hu and Chen 2018; Wang et al. 2021; Zhong et al. 2021).

Transplanted *P.australis* (*Poaceae*) and *A.donax* (*Arundineae*) were taken from a natural wetland in ISA and were weekly irrigated during April 2023 using tap water of 1.5 L for all units (photos can be found in the supplementary materials). The PVC pipes were placed on a metal stand above the ground to collect drained water and were connected to 5-L buckets for the measurement of drained water volume and sampling.

An urban sewage sludge was collected from Beirolas WWTP, Lisbon (213,510 inhabitants) from both primary and secondary stages flowing into a mixed tank. The mixed tank received the primary sludge after thickening and, the surplus activated sludge after air flotation thickening, designated by mixed sludge. Before the feeding phase, 180 L of mixed sludge was transferred to the Horto facility and stored in two 100-L plastic drums under the greenhouse condition. The drums were capped and placed in a shadow to avoid direct exposure of sun lights used for feeding. Dry and volatile solids (DS and VS) of the sludge were 29.44 ± 1.02 g DS L^−1^ and 24.23 ± 0.84 g VS L^−1^ and measured before each feeding, which reduced to 26.58 g DS L^−1^ and 21.56 g VS L^−1^ during the study in the drums. The feeding phase was between May and September 2023 in ten cycles in which units were fed every 2 weeks followed by a 2-week rest. The sludge was characterized before the storage and its average temperature, pH, and electrical conductivity (EC) were 26.6 °C, 6.25, and 1.69 mS cm^−1^, respectively (additional information in the supplementary materials). The following SLRs were tested: 70 kg DS m^−2^ year^−1^ for the WP1 and 5 (SLR_1_), 60 kg DS m^−2^ year^−1^ for the WP2 and 6 (SLR_2_), and 50 kg DS m^−2^ year^−1^ for the WP3, WP7 (SLR_3_) and control units. The unit area was 170 cm^2^, and based on DS content, 70, 60, and 50 kg DS m^−2^ year^−1^ corresponded to 1.766, 1.513, and 1.261 L of mixed sludge; thus, the hydraulic loading rate was 10.4, 8.9, and 7.4 cm. Gholipour et al. (2024b) tested an SLR of 43.5 kg DS m^−2^ year^−1^ for a configuration similar to the WP7 (*Eisenia fetida* and *A.donax*) in which feeding was between May 2022 and May 2023 and then 132 days of final resting until September 2023 was considered. The tested configurations could compare two plant species under higher SLRs in Portugal’s climate as well. To increase the dryness and stabilization, feeding ceased in September 2023, and the units passed 2 months of final resting as suggested in previous studies (Gholipour et al. 2022). To compare the effects of final resting periods in both dry and wet seasons, this study aimed to conduct experiments during different climatic conditions. While our previous study conducted at Beirolas examined the final resting period during the dry season, the current study sought to investigate the same parameter during the wet season in Horto. This deliberate choice allowed for a comparative analysis of how varying seasonal conditions impact the effectiveness of the final resting phase in our experiments and how different weather affects final dry solid and residual sludge quality. Therefore, the final resting period in the present study was specifically scheduled during the wet season to facilitate this comparative analysis between the Horto and Beirolas experiments.

### Plant growth and earthworm dynamics

Detailed morphometric parameters, encompassing both average sizes and densities, were documented to evaluate plant development. A tape measure was used to gauge the distance from the plant’s base, directly in contact with the sludge layer, to their apical part. Plant density was counted as the number of stems in each unit, which is crucial in reflecting the plant population. The measurements were conducted four times during the study including at the time of transplantation, after 2 months of resting, at the last cycle of feeding, and at the end of the study, providing a comprehensive understanding of plant growth and progression over time. Additionally, to measure the aboveground plant biomass accurately, reeds were harvested at the end of the final resting period in November 2023 and dried in a 60 °C oven for 3 days until dry biomass achieved a constant weight.

To study the effect of worms on the systems, a manual sorting process was conducted following a flip and strip test to count the worm population in the whole layers of residual sludge and turf (Gutiérrez-López et al. 2016). The number of worms was counted through a dig in the PVC pipe. In addition, onsite observations like cocoons were registered.

### Physicochemical analyses and sampling

Twice a month, residual sludge was collected to measure DS and VS contents, taken from the top and bottom layers using a core sampler (Stefanakis and Tsihrintzis 2011). A tape measure was sticked to the inner side of each unit to measure the thickness of residual sludge, recorded at the end of the 2-week rest multiplied by each unit area to quantify the volume of residual sludge.

Lab analyses for residual sludge, mixed sludge, and drained water were conducted to determine DS, VS, pH, EC, and temperature. Additionally, pH and EC of the mixed sludge and drained water samples were measured, in situ using a handheld multi-parameter VWR MU 6100 H. Dewatering efficiency was interpreted based on DS, VS, and residual sludge thickness (Gholipour et al. 2022). Samples were collected from drained water on each cycle after feeding was completed. Physicochemical analyses of drained water were conducted for turbidity, chemical oxygen demand (COD), total suspended solids (TSS), total volatile solids (TVS), nitrate nitrogen (NO_3_^−^-N), ammonium nitrogen (NH_4_^+^-N), total kjeldahl nitrogen (TKN), total phosphorous (TP) as well as microbiological parameters such as *Escherichia coli* (*E. coli*), *Fecal coliform* (*F. coli*), and *Salmonella* spp. It was according to the Standard Methods for the Examination of Water and Wastewater (APHA, 2017).

In addition, to assess the content of micro (Fe, Na, B, Mn, and Mo) and macronutrients (N, Ca, P, S, mg and K); microbiological parameters; and heavy metal (HM: Zn, Cr, Cu, Pb, Ni and Cd) in residual sludge, samples were collected from both top and bottom layers after final resting representing the surface and subsurface layers of the accumulated residual sludge quality. To ensure representative samples, residual sludge was uniformly and randomly selected from a designated area and mixed to create a composite sample for each unit in November 2023. For the elemental analysis, inductively coupled plasma (ICP) was utilized (APHA, 2017). Microbiological analyses, encompassing the assessment of *E. coli*, *F. coli*, and *Salmonella* spp, were conducted in November 2023 (photos of drained water samples can be found in the supplementary materials).

### Mass balance, removal efficiency, and water loss estimation

To conduct water balance analysis and estimate evapotranspiration (ET: mm day^−1^), inflows and outflows were recorded. ET was calculated for each cycle according to Eqs. 1 and 2 (Gholipour et al. 2024b):

1 $$\text{WL}={\text{P}}_{\text{r}}+{\text{V}}_{\text{MS}}-{\text{V}}_{\text{RS }(\text{A})}-{\text{V}}_{\text{RS }(\text{B})}-{\text{V}}_{\text{P}}-{\text{V}}_{\text{DW}}$$

2 $$\text{ET}=\frac{\text{WL}}{\text{unit area}}$$

where WL is the water loss (L), P_r_ is the precipitation volume (L), V_MS_ is the water volume (L) in mixed sludge, V_RS (A)_ is the water volume in the residual sludge layer before feeding (L), V_RS (B)_ is the water volume in the residual sludge layer at the end of each resting period (L), V_P_ is the water volume draining to the mesoporous media (L), and V_DW_ is the drained water volume (L). P_r_ was measured through the onsite weather station. V_MS_, V_RS (A)_, and V_RS (B)_ were calculated based on DS content (%) in mixed sludge and residual sludge. The thickness of residual sludge was used to estimate V_RS_ in which an average DS of top and bottom was considered. V_DW_ was measured by directly recording the drained water volume out of each unit. In the estimation of water loss based on Eq. 1, V_P_ was assumed zero as most of the water loss was taken from the residual sludge layer, and at the end of the resting period, V_P_ stayed practically steady and close to zero due to the transpiration and fast drainage (Stefanakis and Tsihrintzis 2011). During each 2-week rest, drained water volume was recorded at different time intervals of 1, 2, 4, 6, and 12 h as well as 1, 2, 7, 10, and 14 days after feeding to account for water percolation rate. In addition, to calculate the removal efficiency, Eq. 3 was used:

3 $$\text{Removal efficiency }(\text{\%})=\frac{\left({\text{V}}_{\text{MS}}\times {\text{Con}}_{\text{in}}\right)-\left({\text{V}}_{\text{DW}}\times {\text{Con}}_{\text{iout}}\right)}{{\text{V}}_{\text{MS}}\times {\text{Con}}_{\text{in}}}\times 100$$

where V_MS_ is the volume of mixed sludge (L), Con_in_ is the parameter concentration in the inlet (mg L^−1^), V_DW_ is the volume of drained water (L), and Con_out_ is the parameter concentration in the outlet (mg L^−1^). To estimate mass release (g) in the inlet, each parameter concentration (mg L^−1^) was multiplied by mixed sludge, and in the outlet, each parameter concentration (mg L^−1^) was multiplied by drained water volumes (L).

### Data analysis

The normality and homogeneity of variance for all datasets were evaluated using the Shapiro–Wilk test and Bartlett’s test, respectively. In instances where normality and homogeneity criteria were not met, differences among units were assessed using the Kruskal–Wallis’s test for one-way analysis of variance, with statistical significance set at a *p*-value of 0.05. Thus, depending on the normality of the datasets, the appropriate post hoc tests were conducted following the initial statistical tests. Specifically, after Kruskal–Wallis’s test, Dunn’s test with Bonferroni correction was used for post hoc analysis, and after ANOVA, Tukey’s Honest Significant Difference (HSD) test was employed to account for multiple comparisons. Statistical significance was conducted where the minimum number of samples was five. All statistical analyses were performed using R Studio. Statistical analysis including minimum, maximum, mean, and standard deviation (SD) were computed.

## Results and discussion

### Meteorological analysis

**Fig. 2:**
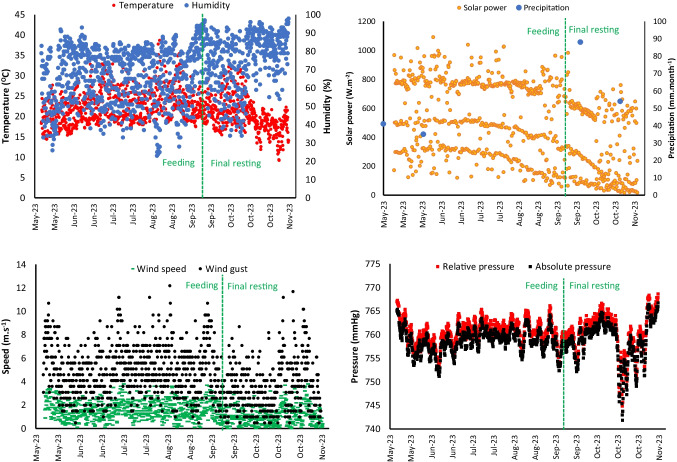
Weather data

Based on the historical dataset (1981–2010), the average annual rainfall of Lisbon is 688 mm, and the absolute temperature varies between 41.2 °C in August and − 1.5 °C in January reported by Instituto Português do Mar e da Atmosfera (IPMA) (Reis et al. 2022). On-site weather station recorded air temperatures ranging from 9.2 to 38.7 °C, accompanied by humidity levels spanning 23 to 98% during the study period. Solar power peaked at 2368 W m^−2^, while average wind speed measured 1.4 m s^−1^ (± 0.5), with gusts reaching 12.2 m s^−1^ (± 4.6) (Fig. 2).

The relative atmospheric pressure measured 761 mmHg, slightly differing from the absolute atmospheric pressure, which was 758 mmHg. Throughout the study period, a total of 218 mm of precipitation was recorded, with 130 mm falling between October and November 15th, 2023. There was no recorded rainfall during June, July, and August. Previous studies also identified a comparable weather data range within the Lisbon district (Andrade and Alcoforado 2008; Reis et al. 2022). According to Schleussner et al. (2020), Lisbon has a Mediterranean climate with mild wet winters and warm dry summers, with relatively low precipitation and temperature variations.

### Plant and earthworm analysis

Plant analysis showed *A.donax* produced higher wet biomass compared to *P.australis*. For SLR_1_ to SLR_3_, the total wet biomass production of *P.australis* was 372, 306, and 265 g in each unit of which 66% was water content by average (8023, 6600, and 5715 g dry m^−2^). *A.donax* wet biomass was 410, 390, and 550 g in each unit with 90% water content by average (2679, 2549, and 3595 g dry m^−2^ for SLR_1_ to SLR_3_). Moreover, higher SLRs resulted in higher biomass production, for instance, SLR_1_ in the WP1 produced 31% higher *P.australis* biomass compared to SLR_3_ in the WP3.

Onsite observation showed by late August 2023, plants in the WP5 and WP6 started getting stressed and dry for the final cycle. Then, they experienced a 2-month period of final rest while the plants exhibited chlorosis. This could show SLRs of 70 and 60 kg DS m^−2^ year^−1^ were above *A.donax* capacity in this specific configuration. The formation of a residual sludge layer on the top of the media possibly created an anaerobic condition minimizing oxygen availability for *A.donax* survival, and hypoxia might increase by the accumulation of sludge in SLRs (Gholipour et al. 2024b). Moreover, water stagnation on the top of the units due to precipitation in early September 2023, could also accelerate the plant stress promoting hypoxia in the residual sludge layer (Loreti and Striker 2020). On the contrary, the WP7 with 50 kg DS m^−2^ year^−1^ continued growing until the end of the experiment. This result is in agreement with the Beirolas pilot, in which *A.donax* was tested for 43.59 kg DS m^−2^ year^−1^.

**Fig. 3:**
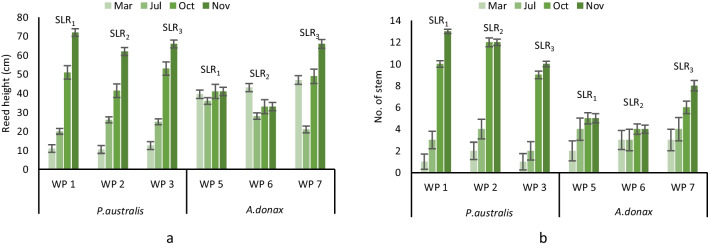
Plant development: height growth (**a**), number of stems (**b**)

Regarding plant development, an average plant growth rate of 12 and 11 cm month^−1^ was registered for *P.australis* and *A.donax*, respectively, with higher growth during the feeding period (Fig. 3). The average number of stems in each unit also increased, from 2 to 11 for *P.australis* and from 3 to 7 stems for *A.donax* (Fig. 3). The higher SLR had a positive effect on the stem number, for example, *P.australis* showed 13 stems for SLR_1_ and 10 stems for SLR_3_. The effect of *A.donax* plant stress is observable for the WP5 and WP6 of which the produced biomass decreased with SLR_1_ and SLR_2_. Yet, the WP7 planted with *A.donax* had higher biomass production although it was fed with SLR_3_.

The number of worms varied in units, and higher SLR resulted in a higher number of worms in which six worms were in the WP1 with SLR_1_ compared to the WP2 and WP3 (SLR_2_ and SLR_3_) that had four and two worms. The number of worms taken was lower than the initial value of 20 worms possibly due to the natural die-off and the effect of seasonal variation (Pezzotti et al. 2021). However, visual observations showed several cocoons were found in the vertical profile of the media and as stated in previous studies, worm reproduction can be ceased during a cold season in which worms leave cocoons for the next dry season reproduction (Pezzotti et al. 2021).

**Fig. 4:**
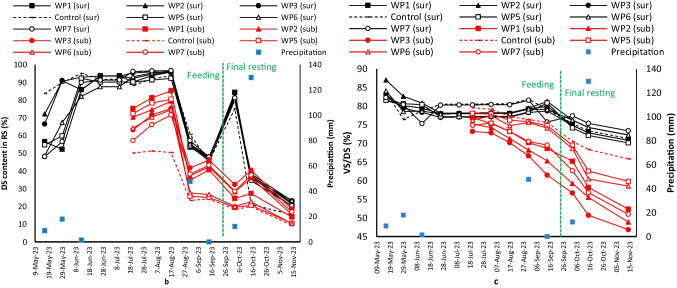
Dewatering efficiency: DS content versus precipitation (**a**), VS/DS content versus precipitation (**b**)

As plants got dried in the WP5 and WP6, the number of worms declined to zero, whereas in the WP7 planted with *A.donax*, there were four worms (Fig. 4, supplementary materials). The absence of worms in the WP5 and WP6 could also be due to oxygen deficiency and exposure to higher SLR. We experimented the effect of seasonal variation on the number of worms in the Beirolas experiment (Gholipour et al. 2024b). It showed that the number of worms increased during the dry season and declined in the wet season especially in October, which is comparable with the current study.

### Dewatering efficiency analysis

**Fig. 5:**
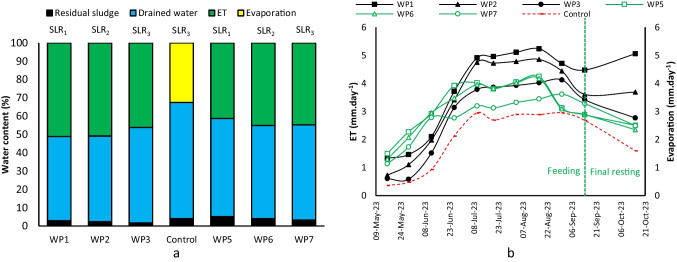
Water balance contribution of each mechanism in water content (**a**) and daily ET and evaporation (**b**)

Residual sludge thickness did not increase until the fifth cycle of feeding (May and July 2023), possibly due to the accumulation of residual sludge within filtration material (Fig. 5, supplementary materials). By the end of the feeding period in September 2023, residual sludge thickness reached around 1 cm for the WP1 to WP3, and around 3 cm for the WP5 to WP7, while the control unit showed 1.5 cm (DS content for all units ≈ 47%). After the final rest, residual sludge thickness was 13, 12.5, 10.5, 14.5, and 14 cm for the WP1 to WP7, respectively, while it was 16.5 cm for the control unit. The accumulated thickness was like WP units achieved in the Beirolas experiment in November 2022, around 10 cm in a WP with *A.donax* (Gholipour et al. 2024b). This could be due to water absorption by the residual sludge layer from the precipitation in September for both Horto and Beirolas experiments after the dry season. Overall, units with *P.australis* resulted in lower residual sludge accumulation compared to units with *A.donax*. It can be attributed to higher ET for *P.australis* and higher decomposition rate of organic matter. In addition, higher SLR led to higher residual sludge accumulation. The WP3 had 36% less accumulation compared to the control unit due to plants and worms while it was 25% for the WP7.

In Beirolas study, an accumulation rate of 0.06, 0.09, 0.05, and 0.1 m year^−1^ was found for the WP, planted, worm, and control units, respectively, in which DS was 70%, while 132 days of final rest were between May to September 2023 (Gholipour et al. 2024b). This was 0.09 m year^−1^ for the STRB unit without worms with a final rest between 25 and 180 days in Mediterranean studies (Stefanakis and Tsihrintzis 2011; El-Gendy and Ahmed, 2020), while it varied between 0.26 and 0.1 m year^−1^ in temperate climate (Gholipour et al 2022). This suggests for minimizing residual sludge volume, and the duration of final rest needs to be longer within a dry season.

Dewatering efficiency indicated DS (Fig. 4a) and VS/DS (Fig. 4b) contents were lower in the subsurface (sub) layers compared to the surface layers (sur) and were significantly different (*p*-value < 0.05). Several factors including rainfall, evaporation, plant transpiration, solar radiation, and wind could influence DS content. During feeding, sur-DS contents of all units were statistically different (*p*-value < 0.05) ranging between 50 and 95%, while during the final rest, they were not statistically different (15 ~ 86%). During feeding, sub-DS contents were different (23 ~ 85%), while after the final rest, it ranged from 9 to 46%. DS contents of the control unit were consistently lower in comparison with the other units, while VS/DS content was higher in the control unit. This indicates the coexistence of plants and worms could increase dewatering efficiency and enhance stabilization.

As it can be seen, the final sub-DS contents of the WP1 to WP7 after 2 months of rest were 14, 15, 18, 10, 10, and 17% (control = 9%), while they were 20, 21, 23, 22, 23, and 20% on the surface (control = 15%), respectively. In terms of sub-VS/DS, WP units showed a 16% reduction (control = 10%) during the final rest; therefore, worms and plants could enhance the stabilization rate by 6%. In addition, higher SLRs like SLR_1_ resulted in lower DS content due to obviously higher feed. In the WP5 and WP6 due to plant wilting, sub-DS contents were like control units. Sub-VS/DS of the WP5 and WP6 units was around 59 and 59% which were higher than other units (52, 49, 47, and 51 for the WP1, WP2, WP3, and WP7) and close to the control unit (65%) (a summary of dewatering efficiency was presented in the supplementary materials).

Overall in the final resting, in units planted with *P.australis* on average, there were 5% higher sub-DS and 3% lower sub-VS/DS values compared to units planted with *A.donax*. The highest DS and lowest VS/DS were achieved in the WP3, planted with *P.australis*, with 50 kg DS m^−2^ year^−1^ (SLR_3_). Furthermore, units planted with *P.australis* and SLR_3_ resulted in 5% higher stabilization compared to SLR_1_.

The final sur-DS obtained in this study for planted units with *P.australis* (41 ~ 46%) is comparable with previous studies that applied units without worms planted with *P.australis*. In temperate and tropical climates, sur-DS was from 33 to 40%, 45% in arid climates, and 30% for a study in polar climates (Gholipour et al. 2022; Dodane et al. 2011; Gagnon et al. 2013). In tropical climates, sur-DS ranged between 63 and 59% for WP and planted units in China (Chen et al. 2016; Hu et al. 2020). In another Chinese study, it also varied between 12 and 50% in WP units (Zhong et al. 2021). For an I-STRB in a temperate climate, a sur-DS of 62.5% was reported (Plestenjak et al. 2021).

The final sub-VS/DS of this study varied between 47 and 52% for planted units with *P.australis*, which is comparable with previous studies, which reported 53, 42, and 40% in temperate, tropical, and polar climates, respectively. In a temperate climate, sub-VS/DS ranged between 51 and 79% (Gagnon et al. 2013; Gholipour et al. 2022). In other temperate climates, it was 67%, while it was 52% for tropical studies (Cui et al. 2015). For WP units in tropical climate studies, sub-VS/DS varied between 33 and 42% (Chen et al. 2016; Hu et al. 2020; Zhong et al. 2021). In previous temperate climates, 39, 67, and 52% were reported with final resting of 60, 120, and 365 days, respectively (Gholipour et al. 2022). In this study, a sub-VS/DS of 46.87% was achieved by 60 days of resting which aligns closely with the previous studies mentioned. Overall, the inclusion of worms in the system could potentially enhance stabilization.

Overall, both plant species indicated a promising dewatering performance in this study, while the DS content of the residual sludge was significantly different among units (*p*-value < 0.05). This could be attributed to the extension of the root system of the studied plants affecting dewatering efficiency (Gagnon et al. 2013; Hu et al. 2020). The root structure of both plant species also would influence the aeration within the reed bed as well as the water drainage. Microorganisms on root exudates together with the presence of earthworms potentially impacted the breakdown of sludge components through their digestive processes.

### Water balance analysis

There was a total influent volume (including water from mixed sludge and precipitation) of 18.78 L in the WP1 and WP5, 16.56 L in WP2 and WP6, and 14.35 L in the WP3, WP7, and control (Table 1). A part of the influent volume underwent drained water, and a marginal volume remained in the residual sludge layer.

**Table 1:** Water balance parameters

Units	Mixed sludge fed (L)	DS	Influent from mixed sludge	Precipitation (L)	Total influent	Total drained water	Water content in residual sludge (L)	Water loss
		(L)	(L)		(L)	(L)		(L)
WP1	17.66	0.43	17.23	1.56	18.78	9.57	0.31	8.91
WP2	15.38	0.38	15.01	1.56	16.56	8.66	0.22	7.69
WP3	13.12	0.32	12.79	1.56	14.35	8.88	0.18	5.30
Control	13.12	0.32	12.79	1.56	14.35	10.86	0.33	3.17
WP5	17.66	0.43	17.23	1.56	18.78	11.34	0.32	7.13
WP6	15.38	0.38	15.01	1.56	16.56	9.50	0.24	6.82
WP7	13.12	0.32	12.79	1.56	14.35	8.92	0.28	5.15

The result of the water balance analysis showed water loss accounted for 47.42, 46.40, 36.89, 37.95, 41.15, and 35.88% of the total influent for the WP1 to WP7 units, while it was only 22.05% for the control unit. Thus, units planted with *P.australis* had higher water loss compared to units planted with *A.donax*; for instance, the WP1 had 10% (1.77 L) higher water loss compared to the WP5 with a similar SLR of 70 kg DS m^−2^ year^−1^. This could be due to the higher ET rate of *P.australis* and a higher number of worms enhancing dewatering. In terms of drained water, the WP5 released the highest amount (11.34 L) and the WP2 the lowest (8.66 L).

The water percolation rate was around 0.4 m day^−1^ at the first cycle for the WP1 to WP7, while it was 0.6 m day^−1^ for the control unit. At the last cycle in September, it reduced to 0.073, 0.081, 0.085, 0.067, 0.072, and 0.078 m day^−1^ for the WP1 to WP7, respectively, and the control unit had a water percolation rate of 0.1 m day^−1^. This indicates unit with *P.australis* had a lower water percolation rate due to higher water loss and DS, plant root system, and media biofilm development.

The mechanisms for water loss in WP units were basically through residual sludge, drained water, and ET (Fig. 5a), and likewise, for control units were through residual sludge, drained water, and evaporation. The contribution of residual sludge in water loss in units planted with *P.australis* was lower than units planted with *A.donax* of which 2.88, 2.39, and 1.68% were for the WP1 to WP3 and 5.16, 4.12, and 3.23% for the WP5 to WP7 (control unit = 4.14%).

This behavior can be attributed to the higher DS content in units planted with *A.donax* indicating different ET using *P.australis* and *A.donax*, while SLRs were similar. The higher SLR also increased residual sludge contribution in water loss. The most important pathway of water loss was through drained water for all units, with the control unit showing the highest value of 63%, and the lowest was for the WP1 by 46%. Thus, units with *P.australis* showed lower drained water contribution in water loss compared to units with *A.donax*. Water loss through ET was 51, 50, 46, 41, 45, and 45% for the WP1 to WP7, while the control unit showed a 33% contribution. In the Beirolas experiment, a notable increase in drained water contribution to water loss was observed during the dry season. However, this contribution decreased during the wet season, possibly as a result of the accumulation of a residual sludge layer and the accumulation of increased water content on the surface.

In terms of ET (Fig. 5b), within May and June, the WP5 to WP7 units planted with *A.donax* showed a higher ET rate compared to the WP1 to WP3 planted with *P.australis*. Afterwards, ET of the WP1 to WP3 surpassed ET of the WP5 to WP7 indicating *P.australis* development after 2 months of feeding. The WP1 with SLR_1_ showed the highest ET rate, which was 5.23 mm day^−1^ in August, whereas it was 4.24 mm day^−1^ (SLR_1_) for the WP5. The effect of plant loss in the WP5 and WP6 during August is significant, illustrated by a sudden drop in ET from 4.24 to 2.98 mm day^−1^. At the end of the study, the WP1 indicated 68 and 27% enhancement in ET rate compared to the control unit and the WP2, respectively. This reveals that plants, worms, and SLR were effective factors. In the Beirolas experiment, the contribution of residual sludge layer in water loss was 4.82 and 2.17% for the WP and planted units (with *A.donax*) which is comparable with the present study with 5.16, 4.12, and 3.23% for the WP5 to WP7 (Gholipour et al. 2024b). Water loss through drained water was also 16 and 45% for WP and planted units in the Beirolas study, while it was 52, 51, and 52% for the WP5 to WP7 in the present study.

In total, cumulative ET for the WP1 to WP7 were 10.95, 9.41, 7.85, 8.71, 8.44, and 7.64 L, respectively (control unit = 5.58 L). Reduction in SLR from 70 to 60 kg DS m^−2^ year^−1^ decreased cumulative ET by 14% for the WP1 and WP2 (*P.australis*) while reducing from 60 to 50 kg DS m^−2^ year^−1^ between the WP2 and WP3 led to a 17% reduction, whereas for units planted with *A.donax*, the same change in SLR decreased ET only by 3 and 9%. Maximum ET rate fell in August for Horto, which was 5.23, 4.85, 4.02, 4.24, 4.18, and 3.44 mm day^−1^ for the WP1 to WP7, while the evaporation rate was 2.88 mm day^−1^ (Fig. 5b). This is comparable with ET for the Beirolas study in August (dry season), which was 5.44, 4.16, and 3.27 mm day^−1^ for WP, planted (with *A.donax*) and control units, respectively (Gholipour et al. 2024b). After the last feed for Horto, ET reduced, and then during the final resting in October, it increased by the increase in precipitation. Higher precipitation also triggered higher ET in the Beirolas experiment (Gholipour et al. 2024b).

### Drained water analysis

**Fig. 6:**
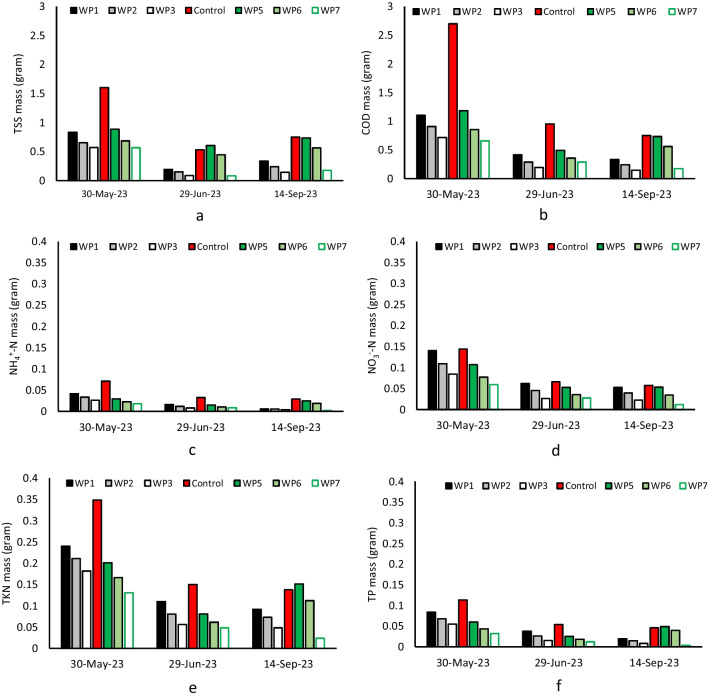
Mass release of TSS (**a**), COD (**b**), TKN (**c**), NH_4_^+^-N (**d**), NO_3_.^—^N (**e**), and TP (**f**)

EC of drained water was 5.82, 6.02, 5.70, 6.07, and 5.68 mS cm^−1^ for the WP1 to WP7 by average (control unit, 6.50 mS cm^−1^), while EC of mixed sludge was 1.69 mS cm^−1^; therefore, an increase in EC of mixed sludge to drained water was observed. This could be attributed to the increase of ions in the drained water due to water loss in the dewatering process through evapotranspiration and earthworms’ hydration needs. Likewise, the pH of drained water was higher than the pH of mixed sludge from 6.25 to 7.4 in all units (no significant difference between units, *p*-value > 0.05) indicating pH neutralization from mixed sludge to drained water. Drained water temperature followed atmospheric temperature variation and was averagely 25 °C for all units (no significant difference between units, *p*-value > 0.05). Drained water quality in all units improved over time and in terms of released masses including TSS, COD, TKN, NH_4_^+^-N, NO_3_^−^-N, and TP parameters, planted units with *P.australis* consistently showed lower mass release (Fig. 6). The gradual improvement of drained water during the feeding period could be due to the system adaptation after a ramp-up phase which was also observed in the previous studies at the end of the dry season (Gholipour et al. 2024a). The drained water contained a low concentration of pollutants after 4 months of feeding which could be due to the formation of residual sludge layer, plant root system, earthworm activities, and reed bed substrates (Stefanakis et al. 2014). Lower SLRs resulted in lower mass release, as expected. The control unit indicated higher mass release compared to other units. The released mass of TSS in May was 0.83, 0.65, 0.57, 0.88, 0.69, and 0.57 g for the WP1 to WP7 (control unit, 1.60 g), while in September, it reduced to 0.19, 0.15, 0.08, 0.61, 0.44, and 0.08 g (control unit, 0.53 g), respectively (Fig. 6a). This corresponded to 77, 77, 85, 31, 35, and 85% reduction for the WP1 to WP7 (control unit, 67%). The residual sludge layer likely contributed to the filtration mechanism in which coarse particles remained on the top of the beds and the fine particles were removed through the combined effects of filter materials and root system (Gholipour et al. 2024a). The root systems could act as natural filters, trapping suspended particles and allowing them to settle which potentially enhanced the reduction of solids.

In addition, COD mass released reduced 70, 74, 80, 38, 34, and 73% (control unit, 72%) from May to September (Fig. 6b) and reached 0.34, 0.24, 15, 0.73, 0.56, and 0.18 g for the WP1 to WP7 (control unit, 0.75 g), respectively. Several mechanisms were effective in the reduction of COD including earthworms, plants, and filter material. Earthworms through their continuous movements within the substrate and residual sludge layer could create micro tunnels accelerating oxygen transmission into the beds (Vymazal et al. 2021). Simultaneously, they broke down residues and utilized organic matter contributing to the treatment of the drained water. Plant root systems could also increase oxygenation creating aerobic conditions favoring oxidation of organic matter (Stefanakis et al. 2014). Ammonium mass release reduced from May to September by 85, 84, 85, 17, 17, and 90% in the WP1 to WP7, while the control unit showed a 16% reduction (Fig. 6c). The higher release masses of ammonium in the WP5 and WP6 by 0.02 and 0.018 g could be due to the plant stress. Nitrate mass release reduced during the study by 62, 64, 73, 50, 55, and 80% in the WP1 to WP7 (control unit, 60%), respectively (Fig. 6d). WP7 (SLR_3_ and *P.australis*) showed a lower release of nitrate compared to the WP3 with a similar SLR and plant, indicating a potential decrease in nitrate availability in drained water using *A.donax* and worms. The general trend of TKN variation was downwards from May to September, but in the WP5 and WP6, there was an increase from June to September attributed to the plant witling in these units (Fig. 6e). The reductions in TKN mass release for the WP1 to WP3 were 62, 65, and 73%, while they were 25, 36, and 82% for the WP5 to WP7 (control unit, 60%), respectively. This shows a 13% difference between the WP3 planted with *P.australis* and the control unit and 21% between the WP7 and the control unit, although TKN mass release increased by the increase in SLR. Several mechanisms stimulated the variations in nitrogen elements in drained water as it could be absorbed in various forms. The aerobic and anaerobic zones created by the root system and earthworm activity supported the nitrification and denitrification processes, converting ammonia to nitrate and then to nitrogen gas, thus reducing nitrogen levels (David et al. 2023).

In terms of nitrate removal efficiency from mixed sludge to drained water, the WP7 (planted with *A.donax*) showed 9% higher efficiency compared to the WP3 with a similar SLR planted with *P.australis*. A similar trend also occurred in case of phosphorous although all units showed over 99% reduction owing to the entrapment of particulate phosphorous on the top of units and the filtration mechanism (Tan et al. 2017). Phosphorous reduction continued after the ramp-up phase and the units planted with *P.australis* showed by average 25% higher removal efficiency compared to the units planted with *A.donax* while *A.donax* was more effective in the removal of nitrogen elements. Phosphorus possibly incorporated into plant biomass, while microorganisms in the rhizosphere contributed to phosphorus mineralization and transformation. This could also make it more bioavailable for plant uptake and, consequently, reducing TP in the drained water (Gholipour et al. 2024a). The effect of SLR was also shown with higher SLR increasing the release of phosphorous.

Nevertheless, WP units were effective in improving drained water quality, particularly in comparison with the control unit, drained water would require a post-treatment stage to comply with the standard limits for water reuse (Decreto-Lei nº 119/2019). Although, NH_4_^+^-N concentrations of the WP1, WP2, WP3, and WP7 were under the limit value of 10 mg L^−1^. *E.coli* limit for class B is 100 CFU mL^−1^, which was met by the WP1, WP2, WP3, WP6, and WP7 units. Overall, units with lower SLRs showed lower concentrations. A summary of drained water quality from the last cycle of feeding (September 2023) in the dry season of the present study and the Beirolas experiment (September 2022) is presented in the supplementary materials for both plant species. They are expressed in concentration and compared with the quality requirements for water reuse.

### Residual sludge analysis

**Fig. 7:**
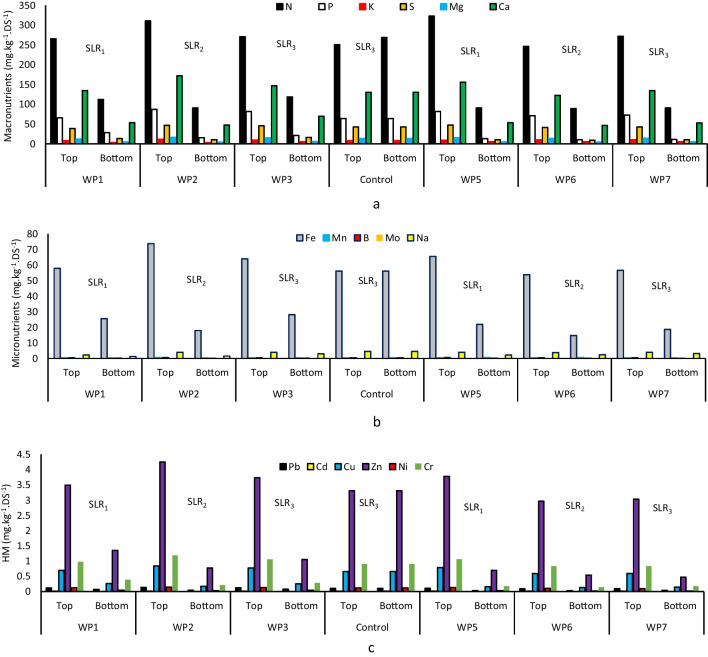
Residual sludge macronutrients (**a**), micronutrients (**b**), and HM (**c**)

Macro and micronutrients and heavy metal (HM) were detected in the final residual sludge. Macronutrient concentration was greater in top layers for the WP1 to WP7 by 524, 645, 569, 633, 504, and 546 mg kg_DS_^−1^ compared to bottom layers at 215, 171, 236, 177, 165, and 175 mg kg_DS_^−1^ (Fig. 7a) which was 509 and 527 mg kg_DS_^−1^ for the top and bottom layers in the control unit, respectively. This could be due to the combined effect of plants and worms reducing elements on the bottom layers while the entrapment of particulate matters on the top of the units increased the availability of the elements. Cumulative mass of macronutrients was 60, 73, and 59% lower than in the bottom for the WP1 to WP3 planted with *P.australis*, while it was 72, 68, and 68% for the WP5 and WP7 planted with *A.donax*. WP units planted with *P.australis* indicated a potentially lower macronutrient percentage compared to WP units planted with *A.donax*. The extensive root system of *P.australis* could be influential in the reduction of the macronutrient. The WP5 and WP6 lost plants and worms, which could also influence macronutrients availability in the bottom layers. In addition, these differences can be attributed to several underlying mechanisms within the residual sludge layers, including root exudates and the activities of microorganisms and earthworms (Edwards and Arancon 2022; Saeed et al. 2022). The root system could possibly promote efficient degradation of organic matter and nutrient cycling (Loreti and Striker 2020). The elemental order of macronutrient significance was N > Ca > P > S > Mg > K of which nitrogen was more abundant, while calcium was another significant fraction. The proportion of NPK was averagely 29:8:1 on the top and 23:4:1 on the bottom for WP units (31:8:1 on the top and 34:8:1 on the bottom for the control unit). The elemental order of macronutrients aligned with the feeding sludge in which N was also the most abundant component.

The effect of higher SLR in the WP1 did not make any clear difference in the cumulative mass of macronutrients compared to the lower SLRs in the WP2 and WP3; however, the WP5 with SLR1 showed higher macronutrient availability compared to the WP6 and WP7 units. Since the final resting started in September and ended in November, this could be the effect of infiltration through precipitation in the wet season; thus, nutrients leached frequently to the drained water and the vertical profile of the residual sludge layer indicated a uniform concentration of nutrients.

In addition, the top layers of residual sludge for WP units had more micronutrients than the bottom layers (Fig. 7b) except in the control unit. This indicates the influence of worms and plants, reduced micronutrient availability in the bottom layers. *E.fetida* could also affect the bottom layers of the residual sludge through bioturbation improving the aeration and mixing of the sludge (Pezzotti et al. 2021). This could potentially facilitate the decomposition of organic matter and the release of nutrients. The synergistic effects of plant roots and earthworms’ activity could potentially create optimal conditions for higher organic matter degradation and nutrient removal, enhancing the overall stabilization and nutrient composition of the residual sludge (Edwards and Arancon 2022; Gutiérrez-López et al. 2016; Pezzotti et al. 2021). The elemental order of micronutrient significance was Fe > Na > B > Mn > Mo in which Fe represented 95% of the cumulative mass, which could be due to the addition of ferric chloride in Beirolas WWTP for flocculation. In terms of the SLR effect, the behavior of micronutrient availability was like macronutrient variations, namely, higher SLR did not increase cumulative mass, particularly in the WP1 to WP3, while the WP5 indicated a higher presence of macronutrients compared to the WP6 and WP7. Plants also did not make a significant increase or decrease in macronutrient availability. Based on Fig. 7c, HM elements were also present in residual sludge by a significant order of Zn > Cr > Cu > Pb > Ni > Cd. The top layers had also a higher availability of HM compared to the bottom layers possibly due to plants and worms. This indicates HM potentially varied due to various mechanisms in the presence of plants and worms. Bioaccumulation, metal binding, improved aeration of residual sludge, stimulation of microbial activity, biological uptake, and enhancement of residual sludge structure could be other factors (Chen and Hu 2019). Worms transform toxic elements like HM into less toxic forms and immobilize them within the residual sludge particles (Ma et al. 2020) (summary of HM in bottom layers, and insights from previous research were presented in the supplementary materials).

Looking into the previous studies shows that a longer duration of final resting would increase the availability of HM due to residual sludge layer compaction during dehydration. The operational conditions of this study are comparable with Chen and Hu (2019) in tropical climate, while the results are different in which 60 days of final resting in units with plants (*P.australis*) and worms were assessed.

Regarding microbial contamination, it was found after 60 days of final resting, *E.coli* and *Fecal coliform* reduced by 99.9% across all units (4 and 3 log removals; *E.coli* and *Fecal coliform* of mixed sludge were 7.5E + 04 and 5.3E + 04 and *Salmonella* was present). Both *E.coli* and *Fecal coliform* values dropped below 1000 CFU 100 mL^−1^ for WP units, while in the control unit, they were 4200 and 1600 CFU 100 mL^−1^, respectively (national and international standard limits were shown in the supplementary materials).

HM concentration fell below all standard limits especially for Portugal (Decreto Lei 276/2009), which could be due to the shorter period of the study and lower accumulation of HM in comparison with previous studies. Thus, WP units met the Portuguese limits (Decreto Lei 276/2009) mandating less than 1000 CFU 100 mL^−1^. Enhanced oxygenation by worms within the residual sludge layer and accelerated competition for nutrients and organic matter affecting the microbial community could improve disinfection efficiency in WP units and the effect on plant root system (Wang et al. 2019). *Salmonella* was present in control, the WP5 and WP6 units while it was absent in the WP1, WP2, WP3, and WP7 units. This could be due to the plant and worm losses in the WP5 and WP6. Thus, the presence of plants and worms could potentially be effective in *Salmonella*. According to Portuguese law (Decreto Lei 276/2009), the absence of *Salmonella* is required in a 50 g sample. Therefore, the WP1, WP2, WP3, and WP7 units complied with this regulatory requirement. Overall, residual sludge obtained from the WP1, WP2, WP3, and WP7 units met the standard limits; however, it should be checked for other contaminations, particularly emerging pollutants; thus, an additional disinfection stage for a safe reuse is suggested. Overall, the residual sludge could be recovered for reuse in case local regulations for sludge reuse are met regarding various pollutants including biological, chemical, and microbial contaminations. Therefore, further studies and field trials are recommended to optimize application practices and ensure the long-term benefits and safety of using sludge as a soil amendment.

## Conclusions

Two plant species, namely *P.australis* and *A.donax* combined with *Eisenia fetida*, were tested in STRB technology under control condition in Portugal. SLR of 50, 60, and 70 kg DS m^−2^ year^−1^ was studied in the WP and C units fed with mixed sludge. Units with *A.donax* lost their plants and worms around the last cycle when SLR was above 60 kg DS m^−2^ year^−1^. Higher SLR yielded higher biomass and elevated numbers of worms, particularly for *P.australis*. Unit with *P.australis* fed by 70 kg DS m^−2^ year^−1^ produced 31% more biomass compared to 60 kg DS m^−2^ year^−1^. Precipitation during the final rest caused an increase in the thickness of residual sludge to more than 10 cm for all WP units (C unit, 16.5 cm) due to the reduction in DS content. Residual sludge accumulation increased by higher SLRs. Sur-DS content was 7% greater than sub-DS (no significant difference between units), while sub-VS/DS was averagely 15% less than sur-VS/DS. VS/DS reduced during a 2-month of final resting by 13%, averagely. Water balance analysis showed water loss increased with higher SLR, and units with *P.australis* showed 20% higher water loss compared to units with *A.donax*. Water loss through absorbance in the residual sludge layer was 2.88% for *P.australis* and 5.16% for *A.donax*, both with SLR of 70 kg DS m^−2^ year^−1^. Furthermore, water loss was 46 and 53% through drained water, and 51 and 41% through ET for *P.australis* and *A.donax*, respectively. A 10 kg DS m^−2^ year^−1^ greater SLR resulted in 14% higher ET for both plants and SLRs. The unit with *P.australis* and SLR of 70 kg DS m^−2^ year^−1^ showed the highest ET by 5.23 mm day^−1^, while the unit with *A.donax* and similar SLR showed 4.24 mm. Drained water quality ought to be analyzed in terms of both mass release and pollution concentration allowing a correct assessment of total pollutants discharged. Drained water improved over time, and units with *P.australis* released a lower mass of COD, TSS, TKN, NH_4_^+^-N, and TP compared to units with *A.donax*, and in addition, higher SLR resulted in greater mass release. Drained water quality did not meet standard limits for water reuse. Residual sludge quality showed top layers had a higher availability of nutrients and HM compared to bottom layers, while the availability of them was not significantly different between units. The elemental order of macronutrients showed a notable hierarchy with N > Ca > P > S > Mg > K, while for micronutrients, the sequence was Fe > Na > B > Mn > Mo. Heavy metal availability adhered to standard limits, ranking Zn > Cr > Cu > Pb > Ni > Cd. The incorporation of worms into STRB has the potential to improve dewatering efficiency, significantly. However, it is crucial to analyze carefully factors such as SLR, operational modes, and the specific characteristics of the sludge when selecting the appropriate season and plants for optimal results.

## Supplementary Information

Below is the link to the electronic supplementary material.

Supplementary file1 (DOCX 8085 kb)

## Data Availability

The data that support the findings of this study are available from the authors, but restrictions apply to the availability of these data, and so are not publicly available. Data are, however, available from the authors upon reasonable request.

## Abbreviations

NH_4_^+^-N: Ammonium nitrogen
*A.donax*: *Arundo donax*
COD: Chemical oxygen demand
DS: Dry solids
E-STRB: Electro-sludge treatment reed bed
EC: Electrical conductivity
*E. coli*: *Escherichia coli*
ET: Evapotranspiration
*F. coli*: *Fecal coliform*
I-STRB: Intensified-sludge treatment reed bed
ISA: Instituto Superior de Agronomia
IPMA: Instituto Português do Mar e da Atmosfera
ICP: Inductively coupled plasma
NBS: Nature-based solutions
NO_3_^—^N: Nitrate nitrogen
*P. australis*: *Phragmites australis*
STRB: Sludge treatment reed bed
SLR: Sludge loading rate
Sub: Subsurface
Sur: Surface
SD: Standard deviation
TSS: Total suspended solids
TVS: Total volatile solids
TKN: Total kjeldahl nitrogen
TP: Total phosphorous
VS: Volatile solids
WWTP: Wastewater treatment plant
W-STRB: Worm-sludge treatment reed bed
WP: Worm-planted
