# Supplementary material for: A comparative study of worm-sludge treatment reed bed planted with Phragmites australis and Arundo donax in the Mediterranean region
Source: Environ Sci Pollut Res Int. 2024 Aug 8;31(39):51551–67. doi: 10.1007/s11356-024-34632-9 (PMC11374837; doi:10.1007/s11356-024-34632-9)
Supplement: Supplementary file 1 — Supplementary file1 (DOCX 8085 kb) [file 11356_2024_34632_MOESM1_ESM.docx]

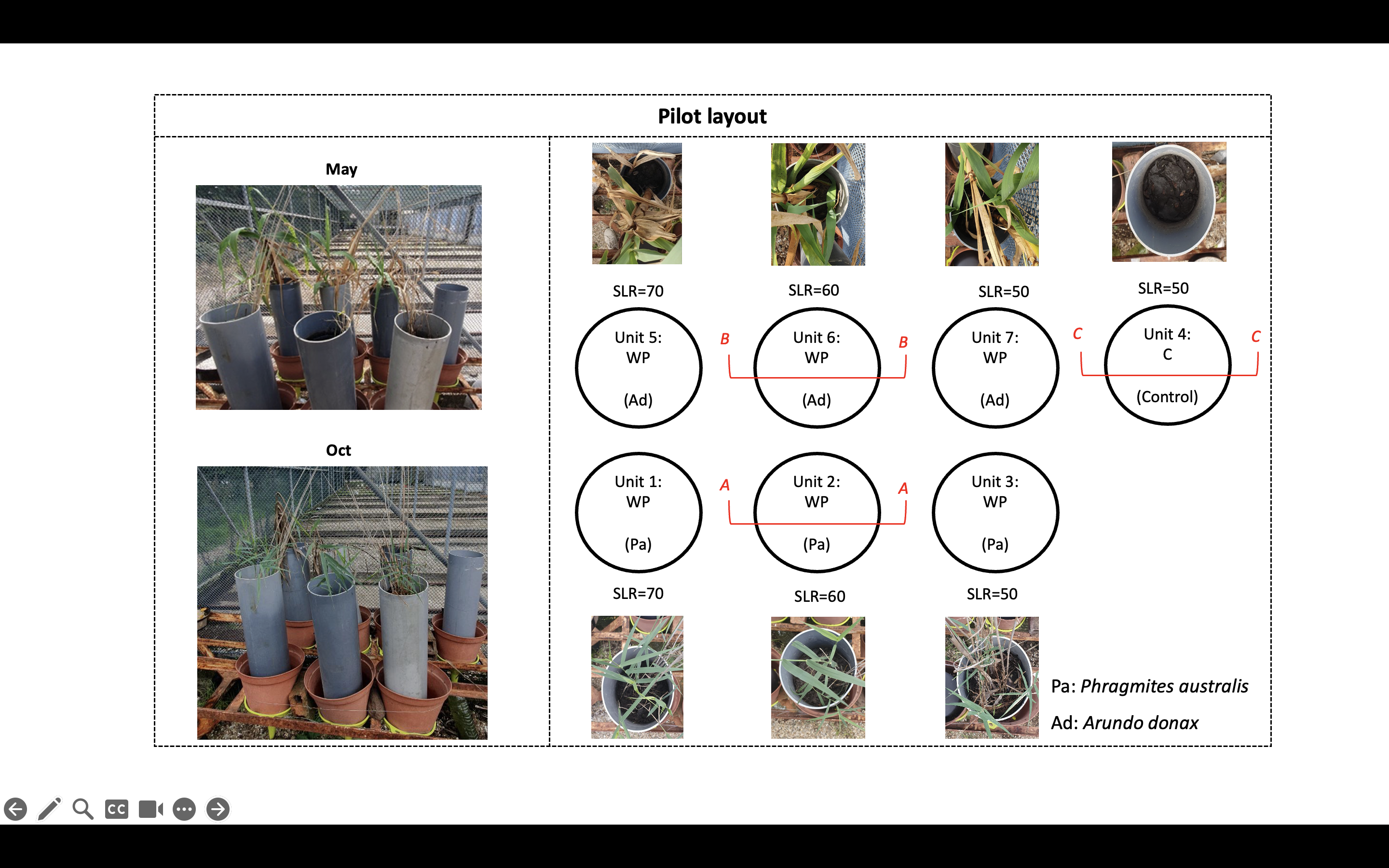


Figure 1. Extensive configuration of the study

Table 1. Mixed sludge characteristics

| **Parameter** | **Unit** | **Value** |
| --- | --- | --- |
| pH | - | 6.25 |
| Electrical Conductivity (EC) | ms.cm^-1^ | 1.69 |
| Total Dissolved Solids (TDS) | mg/l | 1078 |
| Temperature | Centigrade | 26.60 |
| Dry Solid (DS) | g.L^-1^ | 29.44 |
| Volatile Solid (VS) | g.L^-1^ | 24.23 |
| VS/DS | % | 82 |
| TKN | mg.L^-1^ | 1244 |
| N-NH4^+^ | mg.L^-1^ | 334 |
| COD | mg.L^-1^ | 21121 |
| BOD | mg.L^-1^ | 5184 |
| TN | mg.kg^-1^.DS^-1^ | 79456 |
| TP | mg.kg^-1^.DS^-1^ | 149 |
| Na | mg.kg^-1^.DS^-1^ | 40.43 |
| K | mg.kg^-1^.DS^-1^ | 43.28 |
| Ca | mg.kg^-1^.DS^-1^ | 228 |
| Mg | mg.kg^-1^.DS^-1^ | 30.71 |
| S | mg.kg^-1^.DS^-1^ | 62.95 |
| Fe | mg.kg^-1^.DS^-1^ | 81.45 |
| Cu | mg.kg^-1^.DS^-1^ | 0.79 |
| Zn | mg.kg^-1^.DS^-1^ | 3.96 |
| Mn | mg.kg^-1^.DS^-1^ | 0.69 |
| B | mg.kg^-1^.DS^-1^ | 0.66 |
| Mo | mg.kg^-1^.DS^-1^ | 0.04 |
| Cr | mg.kg^-1^.DS^-1^ | 1.15 |
| Ni | mg.kg^-1^.DS^-1^ | 0.15 |
| Cd | mg.kg^-1^.DS^-1^ | 0.01 |
| Pb | mg.kg^-1^.DS^-1^ | 0.12 |

Table 2. Plan of feeding

| **Cycle** | **Date** |
| --- | --- |
| Irrigation with tap water | 17/04/2023 |
|  | 24/04/2023 |
|  | 08/05/2023 |
|  |  |
| 1 | 16/05/2023 |
| 2 | 29/05/2023 |
| 3 | 12/06/2023 |
| 4 | 26/06/2023 |
| 5 | 10/07/2023 |
| 6 | 20/07/2023 |
| 7 | 03/08/2023 |
| 8 | 17/08/2023 |
| 9 | 31/09/2023 |
| 10 | 14/09/2023 |


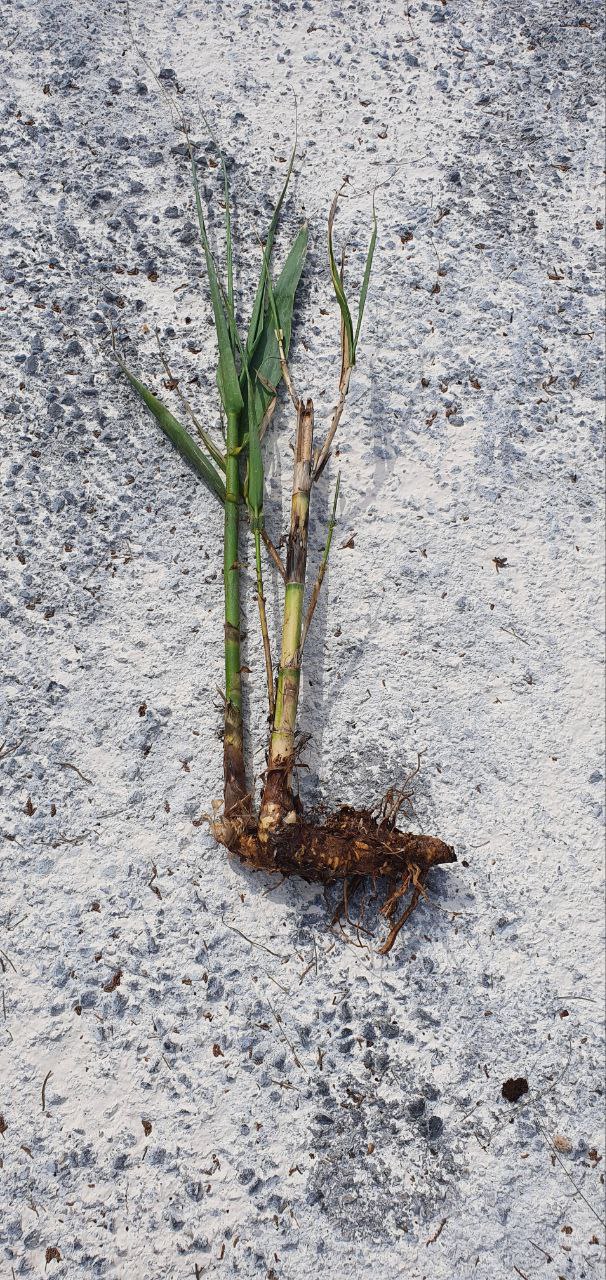

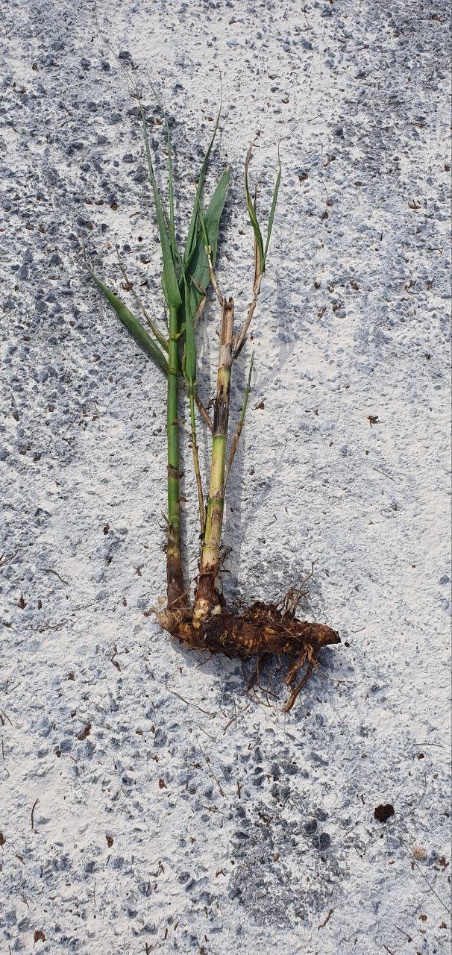


Fresh shoot

Lenght of base

Lenght of root

Lenght of stem

Stem

Lenght of leaf


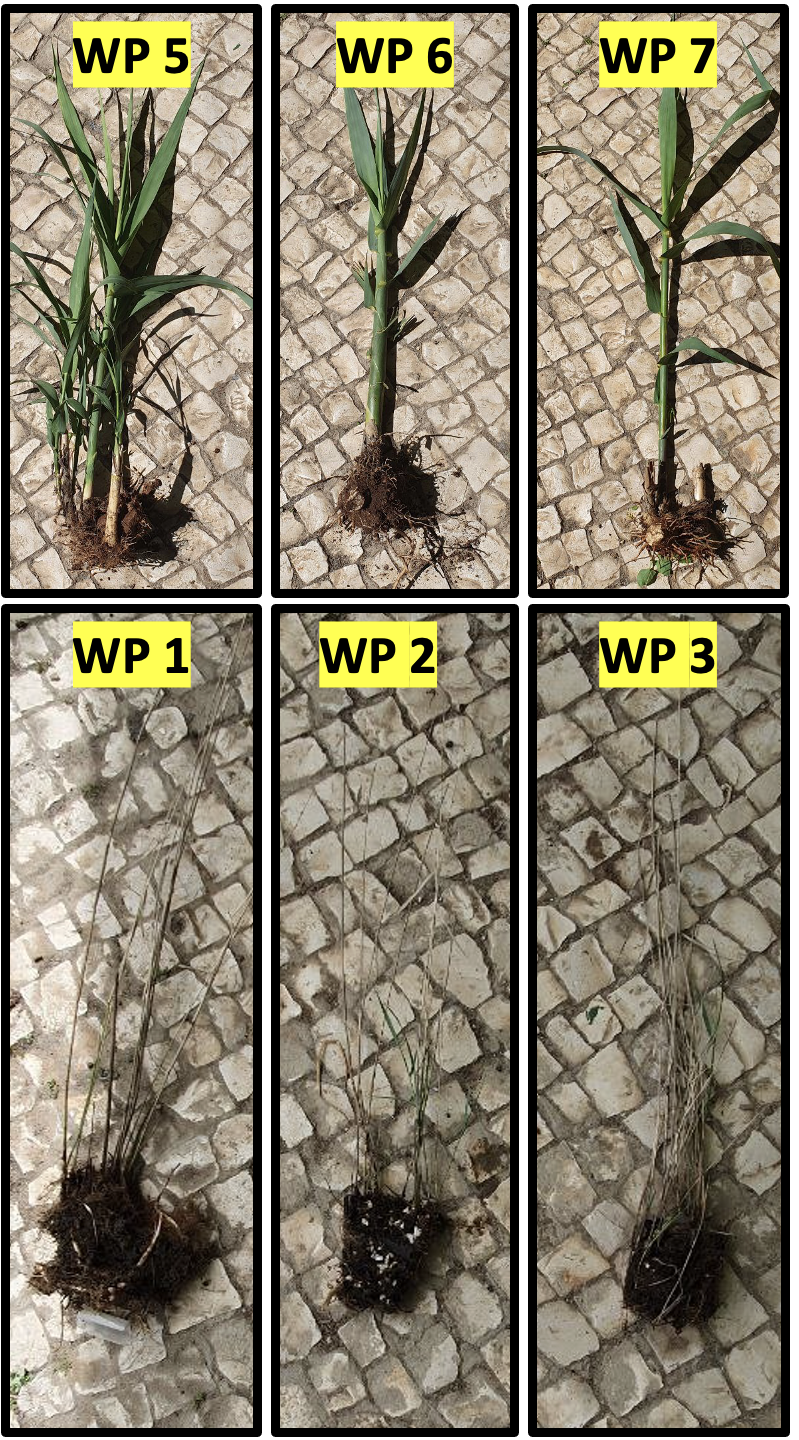


Figure 2. Visualization of plant specifications (for Arundo donax)


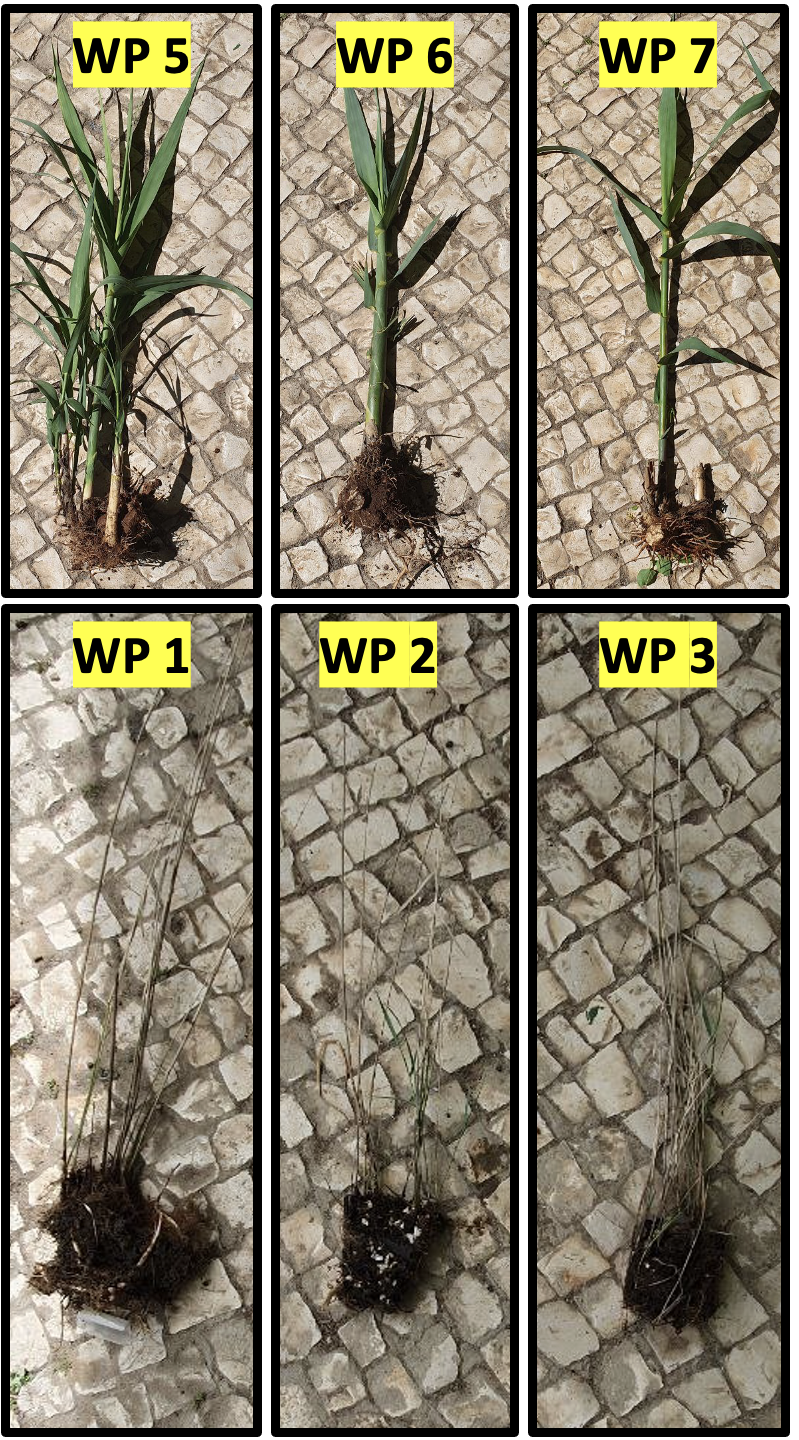

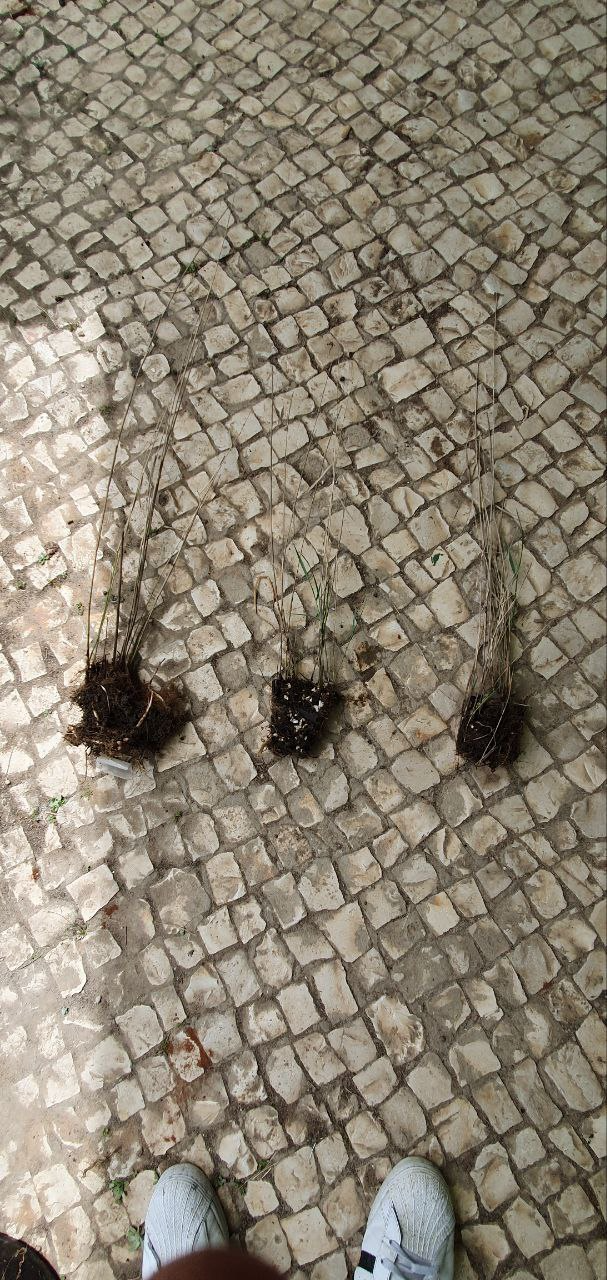


Lenght of root

Lenght of stem

Stem

Figure 3. Visualization of plant specifications (for Phragmites australis)

Table 3. Initial plant specifications

|  | **WP 1** | **WP 2** | **WP 3** | **WP 5** | **WP 6** | **WP 7** |
| --- | --- | --- | --- | --- | --- | --- |
| Weight (g) | 402 | 348 | 383 | 1023 | 830 | 900 |
| No of stems | 2 | 1 | 1 | 3 | 0 | 3 |
| Stem lengths (cm) | 11 | 10,5 | 12,5 | 39,5 | 43 | 47 |
| Platform length (cm) | - | - | - | 10 | 13 | 11 |
| Root length (cm) | 10 | 9 | 13 | 14,5 | 8 | 13,5 |

Figure 4. Eisenia fetida variation

Table 4. Average DS and VS/DS values for all units during the feeding period and the final rest (%).

| **Units** |  | **SLR (kg.DS.m^-2^-year^-1^)** |  | **Plant specie** |  |  | **Feeding period** | | | |  |  | **Final rest** | | | |
| --- | --- | --- | --- | --- | --- | --- | --- | --- | --- | --- | --- | --- | --- | --- | --- | --- |
|  |  |  |  |  |  | Surface | |  | Subsurface | |  | Surface | |  | Subsurface | |
|  |  |  |  |  |  | DS | VS/DS |  | DS | VS/DS |  | DS | VS/DS |  | DS | VS/DS |
| WP1 |  | 70 |  | *P.australis* |  | 47 | 80 |  | 41 | 69 |  | 20 | 71 |  | 14 | 52 |
| WP2 |  | 60 |  | *P.australis* |  | 48 | 81 |  | 42 | 65 |  | 21 | 72 |  | 15 | 49 |
| WP3 |  | 50 |  | *P.australis* |  | 48 | 79 |  | 46 | 62 |  | 23 | 71 |  | 18 | 47 |
| Control |  | 50 |  | - |  | 45 | 79 |  | 24 | 76 |  | 15 | 71 |  | 9 | 66 |
| WP5 |  | 70 |  | *A.donax* |  | 46 | 81 |  | 25 | 74 |  | 22 | 70 |  | 10 | 60 |
| WP6 |  | 60 |  | *A.donax* |  | 48 | 79 |  | 27 | 74 |  | 23 | 72 |  | 10 | 59 |
| WP7 |  | 50 |  | *A.donax* |  | 48 | 76 |  | 43 | 70 |  | 20 | 73 |  | 17 | 60 |

Figure 5. RS thickness development


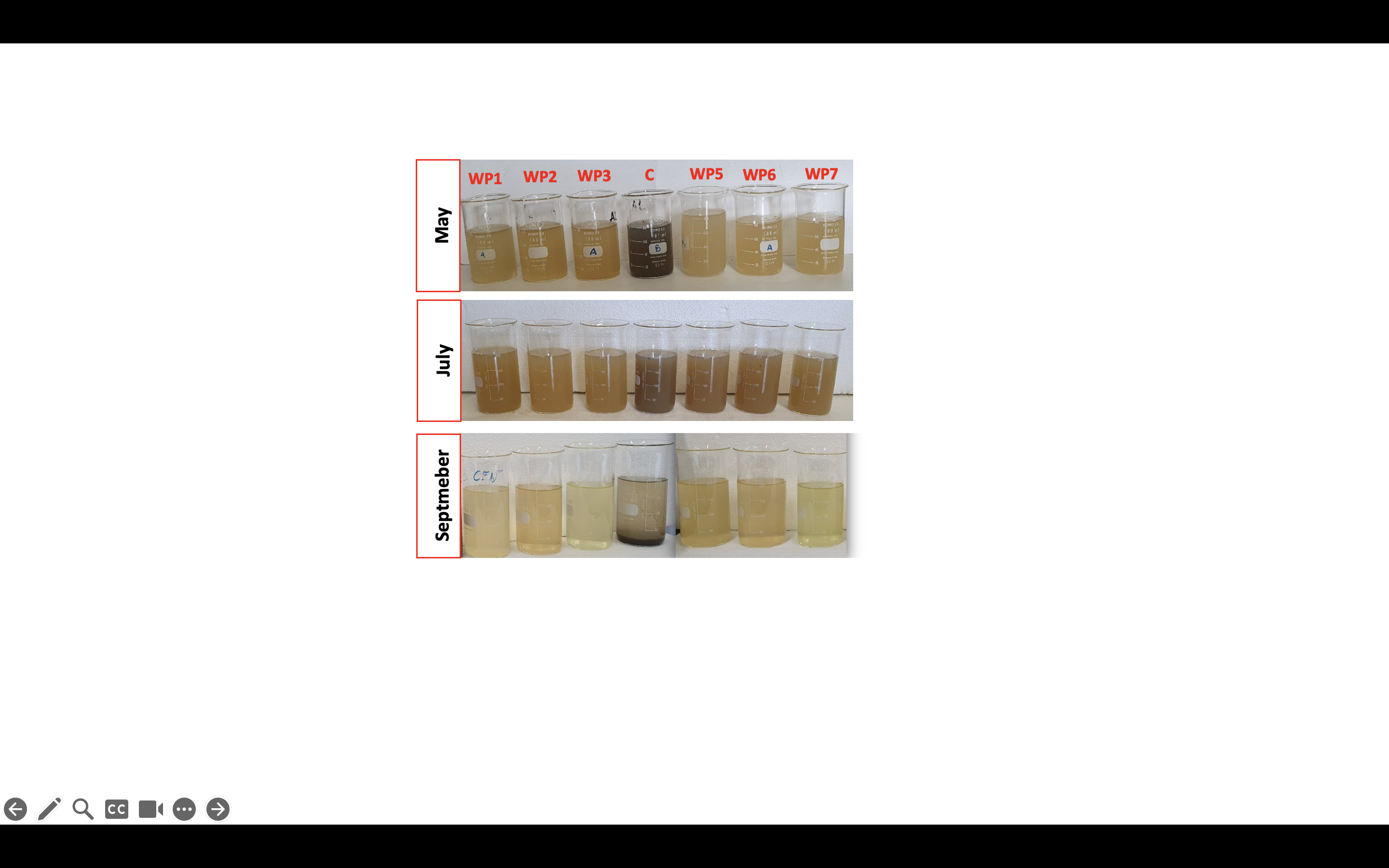


Figure 6. DW samples during the study

Table 5. DW quality for Horto and Beirolas studies

| **Parameter** | **Unit** | **MS: Horto** |  | **Horto** | | | | | | |  | **Beirolas** | | | |  | **Portuguese law for water reuse (Decreto-Lei n.o 119/2019)** |
| --- | --- | --- | --- | --- | --- | --- | --- | --- | --- | --- | --- | --- | --- | --- | --- | --- | --- |
|  |  |  |  | WP1 | WP2 | WP3 | C | WP5 | WP6 | WP7 |  | WP | P | W | C |  |  |
| pH |  | 6.25 |  | 6.56 | 6.66 | 6.89 | 6.46 | 6.67 | 6.77 | 6.87 |  | 6,53 | 6,99 | 7,03 | 7,22 |  |  |
| EC | µs.cm^-1^ | 1,69 |  | 4.56 | 4.18 | 3.89 | 6.10 | 5.03 | 4.84 | 4.32 |  | 8,56 | 6,45 | 5,09 | 3,91 |  |  |
| Temperature | Centigrade | 26.60 |  | 21.50 | 21.60 | 21.50 | 21.45 | 21.30 | 21.20 | 21.50 |  | 18,07 | 18,55 | 17,40 | 18,38 |  |  |
| DS | g.L^-1^ | 29.44 |  |  |  |  |  |  |  |  |  |  |  |  |  |  |  |
| VS | g.L^-1^ | 24.23 |  |  |  |  |  |  |  |  |  |  |  |  |  |  |  |
| Turbidity | NTU |  |  | 168 | 146 | 132 | 257 | 235 | 225 | 151 |  | 130 | 110 | 120 | 102 |  |  |
| TSS | mg.L^-1^ |  |  | 207 | 167 | 127 | 589 | 433 | 405 | 178 |  | 94 | 172 | 120 | 117 |  | ≤10 class A  ≤35 class B |
| TVS | mg.L^-1^ |  |  | 170 | 143 | 104 | 480 | 396 | 385 | 146 |  | 79 | 127 | 109 | 99 |  |  |
| COD | mg.L^-1^ | 21121 |  | 356 | 267 | 217 | 834 | 524 | 510 | 378 |  | 480 | 485 | 296 | 438 |  |  |
| TN | mg.kg^-1^.DS^-1^ | 79456 |  |  |  |  |  |  |  |  |  |  |  |  |  |  |  |
| TKN | mg.L^-1^ | 1244 |  | 97 | 81 | 72 | 153 | 108 | 102 | 51 |  | 99 | 159 | 43 | 200 |  | 15 |
| NH_4_^+^-N | mg.L^-1^ | 334 |  | 6.50 | 6.00 | 5.70 | 32.10 | 17.50 | 16.80 | 4.10 |  | 0,4 | 4 | 5 | 5 |  | 10 |
| NO_3_^-^-N | mg.L^-1^ |  |  | 56 | 44 | 34 | 63 | 38 | 31 | 25 |  | 97 | 72 | 73 | 72 |  |  |
| TP | mg.kg^-1^.DS^-1^ | 149 |  | 21 | 16 | 12 | 51 | 35 | 36 | 7 |  | 99 | 159 | 43 | 200 |  | 5 |
| *Salmonella* | Present/Absent: P/A | P |  | P | P | A | P | P | P | P |  |  |  |  |  |  | A |
| Total Coliform | MPN.100mL^-1^ |  |  | <1600 | <1600 | <1600 | <1600 | <1600 | <1600 | <1600 |  |  |  |  |  |  |  |
| *E.coli* | CFU.mL^-1^ | $8.2\times{10}^{4}$ |  | 100 | 70 | 10 | 550 | 120 | 100 | 30 |  |  |  |  |  |  | ≤10 class A  ≤100 class B  ≤1000 class C  ≤10^4^ class D |
| Fecal coliform | CFU.mL^-1^ |  |  | 1200 | 550 | 65 | 2000 | 1500 | 900 | 120 |  |  |  |  |  |  |  |

Table 6. A summary of HM for the bottom layers in comparison with previous studies

| **Studies and legislations** | | **HM (mg.kg^-1^DS^-1^)** | | | | | | **Final resting duration (day)** | **SLR (kg.DS.m^-2^.year^-1^)** | **Plant** | **Climate** |
| --- | --- | --- | --- | --- | --- | --- | --- | --- | --- | --- | --- |
|  |  | **Zn** | **Cr** | **Cu** | **Pb** | **Ni** | **Cd** |  |  |  |  |
|  |  |  |  |  |  |  |  |  |  |  |  |
| Present study | WP1 | 1,35 | 0,38 | 0,26 | 0,07 | 0,04 | 0,00 | 60 | 70 | *P.australis* | Temperate 2 |
|  | WP2 | 0,77 | 0,21 | 0,17 | 0,04 | 0,04 | 0,00 |  | 60 | *P.australis* |  |
|  | WP3 | 1,05 | 0,28 | 0,25 | 0,07 | 0,05 | 0,00 |  | 50 | *P.australis* |  |
|  | Control | 3,31 | 0,90 | 0,66 | 0,10 | 0,12 | 0,01 |  | 50 | - |  |
|  | WP5 | 0,70 | 0,17 | 0,15 | 0,04 | 0,03 | 0,00 |  | 70 | *A.donax* |  |
|  | WP6 | 0,54 | 0,14 | 0,13 | 0,03 | 0,02 | 0,00 |  | 60 | *A.donax* |  |
|  | WP7 | 0,47 | 0,17 | 0,14 | 0,04 | 0,02 | 0,00 |  | 50 | *A.donax* |  |
|  |  |  |  |  |  |  |  |  |  |  |  |
| Previous studies | Stefanakis and Tsihrintzis, 2012b | 396 | 183 | 90 | 57 | 48 | 6 | 180 | 60 | *P.australis* | Temperate 2 |
|  | Matamoros et al., 2012 | 1361 | 42 | 184 | 68 | 30 | 2 | 365 | 50 | *P.australis* | Temperate 1 |
|  | Kołecka and Obarska-Pempkowiak, 2013 | 107 | 16 | 147 | 10 | 13 | 1 | 14 | 55 | *P.australis* | Temperate 1 |
|  | Caicedo et al., 2015 | 1575 | 64 | 451 | 67 | 39 | - | - | 30 | *P.australis* | Temperate 1 |
|  | Boruszko, 2018 | 1206 | 70 | 154 | 33 | 18 | 3 | 365 | 35 | *P.australis* | Temperate 1 |
|  | Chen and Hu, 2019 | 150 | 12 | 2.1 | 57 | 4 | 1 | 60 | 45 | *P.australis* | Tropical |
|  |  | 141 | 14 | 2.5 | 32 | 4 | 1 |  |  | *T.angustifolia* |  |
|  | Gonzalez-Flo et al., 2023 | 2915 | 26 | 50 | 12 | 31 | 1 | 30 | 65 | *P.australis* | Temperate 2 |
|  | Ma et al., 2023 | 432 | 104 | 112 | 56 | 86 | 4 | - | - | *P.australis* | Tropical |
|  | Nielsen, 2023 | 895 | 32 | 331 | 55 | 39 | - | 365 | 41 | *P.australis* | Temperate 1 |
|  | **Min** | **107** | **12** | **50** | **10** | **13** | **1** | **14** | **30** |  |  |
|  | **Max** | **2915** | **183** | **451** | **68** | **86** | **6** | **365** | **65** |  |  |
|  | **Mean** | **920** | **65** | **170** | **44** | **38** | **3** | **169** | **49** |  |  |
|  | **Standard deviation** | **939** | **57** | **132** | **22** | **24** | **2** | **163** | **13** |  |  |
| *Typha angustifolia: T.angustifolia*  Temperate 1: Cfa, Cfb, Cfc, Cwa, Cwb, Cwc and Temperate 2: Csa, Csb, Csc of the Köppen–Geiger climate classification classified by Gholipour et al, (2022) | | | | | | | | | | | |

Table 7. National and international standard limits for HM

| **Standard limits** | | **HM (mg.kg^-1^DS^-1^)** | | | | | |
| --- | --- | --- | --- | --- | --- | --- | --- |
|  |  | **Zn** | **Cr** | **Cu** | **Pb** | **Ni** | **Cd** |
| EU Directive 86/278/EEC | | 4000 | - | 1750 | 1200 | 400 | 40 |
| Portugal (Decreto Lei 276/2009) | | 2500 | 1000 | 1000 | 750 | 300 | 20 |
| American standard (Part 503,1994) | | 7500 | 1000 | 4300 | 840 | 420 | 85 |
|  | |  |  |  |  |  |  |
| Chinese standard (GB4284-2018) | Class A | 1200 | 500 | 500 | 300 | 100 | 3 |
|  | Class B | 3000 | 1000 | 1500 | 1000 | 200 | 15 |
